# Vascular Alterations Following COVID-19 Infection: A Comprehensive Literature Review

**DOI:** 10.3390/life14050545

**Published:** 2024-04-24

**Authors:** Paschalis Karakasis, Athina Nasoufidou, Marios Sagris, Nikolaos Fragakis, Konstantinos Tsioufis

**Affiliations:** 1 Second Department of Cardiology, Aristotle University of Thessaloniki, Hippokration General Hospital, 54642 Thessaloniki, Greece; pakar15@hotmail.com (P.K.); athinanassi@gmail.com (A.N.); nfrag@auth.gr (N.F.); 2 First Department of Cardiology, National and Kapodistrian University of Athens, Hippokration General Hospital, 11527 Athens, Greece; kptsioufis@gmail.com

**Keywords:** COVID-19, SARS-CoV-2, vascular system, inflammation, endotheliopathy

## Abstract

SARS-CoV-2, the causative agent of the ongoing COVID-19 pandemic, has revealed a broader impact beyond the respiratory system, predominantly affecting the vascular system with various adverse manifestations. The infection induces endothelial dysfunction and immune system dysregulation, creating an inflammatory and hypercoagulable state. It affects both microvasculature and macrovasculature, leading to thromboembolic events, cardiovascular manifestations, impaired arterial stiffness, cerebrovascular complications, and nephropathy, as well as retinopathy—frequently observed in cases of severe illness. Evidence suggests that SARS-CoV-2 infection may result in persistent effects on the vascular system, identified as long-term COVID-19. This is characterized by prolonged inflammation, endotheliopathy, and an increased risk of vascular complications. Various imaging modalities, histopathological studies, and diagnostic tools such as video capillaroscopy and magnetic resonance imaging have been employed to visualize vascular alterations. This review aims to comprehensively summarize the evidence concerning short and long-term vascular alterations following COVID-19 infection, investigating their impact on patients’ prognosis, and providing an overview of preventive strategies to mitigate associated vascular complications.

## 1. Introduction

COVID-19, caused by the coronavirus SARS-CoV-2, first appeared in 2019 and rapidly evolved into a global pandemic, challenging health systems worldwide [1]. Initially, the virus was classified as a primarily respiratory pathogen, capable of inducing acute respiratory distress syndrome (ARDS), similar to the preceding and genetically related SARS virus that caused a milder pandemic in 2002 [2]. However, it quickly became evident that, despite respiratory symptoms remaining prevalent, SARS-CoV-2 exerts its effects on multiple organs (heart, lungs, kidneys, liver, and lymph nodes), with significant implications for the vascular system [3]. This multi-systemic involvement has drawn increasing attention, requiring further understanding and scientific research for the development of medical interventions to improve overall management. 

Vascular involvement in COVID-19 includes a vast spectrum of manifestations, ranging from endothelial injury to vascular damage and coagulation disorders [4,5,6]. The main entrance mechanism for SARS-CoV-2 is through binding to the angiotensin-converting enzyme 2 (ACE2) receptor, which is expressed not only in the respiratory tract, but also in endothelial cells covering blood vessels throughout the human body [7]. The widespread dissemination of the virus initiates a cascade of events culminating in endothelial impairment, inflammation, a hypercoagulable state, and disorders within the immune system [1,8]. This effect can be systematic or more pronounced in a specific organ. In addition to the acute clinical setting, long-term consequences have also been brought to light, further testing healthcare systems and emphasizing the necessity for a better understanding of the virus’s mechanisms. Comprehension of the tangled relationship between the virus and vascular alterations is essential for the development of targeted treatment approaches, improving patient management and moderating the vascular manifestations of COVID-19. Hitherto, many researchers have tried to clarify the interaction of SARS-CoV-2 with the vasculature, aiming to provide new therapeutic strategies and prevention measures [9]. 

In this review, we aimed to investigate the complex relationship between COVID-19 and vascular alterations, aiming to elucidate the pathophysiological mechanisms, clinical manifestations and therapeutic approaches for COVID-19-induced vasculopathy. 

## 2. Mechanisms of Vascular Alterations in COVID-19 

**Figure 1:**
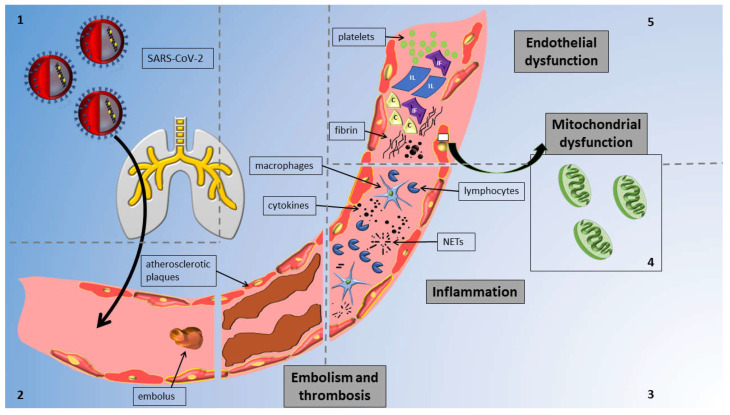
Overview of mechanisms of vascular injury in COVID-19 infection: The figure delineates distinct sections to highlight various mechanisms; notably, these mechanisms collectively impact the vessel and contribute to vascular injury concurrently. Reading anticlockwise, the sections are as follows. Section 1: Virus enters vessels via the respiratory system. Section 2: SARS-CoV-2 induces thrombosis and embolism in affected vessels. Section 3: Virus triggers inflammation via cytokines and neutrophil extracellular traps (NETs) from macrophages and lymphocytes. Section 4: SARS-CoV-2 affects all endothelial cells by causing mitochondrial damage. Section 5: SARS-CoV-2 causes endothelial dysfunction through cells and circulating particles. Abbreviations: IF, interferons; IL, interleukins; C, complement; and NETs, neutrophil extracellular traps.

The mechanisms implicated in vascular alterations attributed to COVID-19 are concisely illustrated in Figure 1. In pathological studies, COVID-19 was linked to critical endothelial alterations including thrombotic occlusions of alveolar capillaries, alveolar remodeling and hyperplasia, the direct or indirect infection of lung endothelial cells (ECs) leading to widespread inflammation, cell oedema, and apoptosis, and the disruption of the normal membrane barrier [10]. 

### 2.1. Endothelial Damage

SARS-CoV-2 has the potential to directly infect ECs and instigate extensive vascular injury by binding with proteins such as ACE2 [11], lymph node-specific intercellular adhesion molecule-3-grabbing integrin (L-SIGN), and transmembrane protease serine 2 (TMPRSS2) [12]. Endothelial injury, impaired lipid metabolism, and hemodynamic disruption, followed by flow-mediated inflammatory changes in the endothelium, are the main steps leading to endothelitis [13]. Inflammation begins when the endothelial cells become activated and secrete adhesion molecules, and the smooth muscle cells secrete chemokines and chemoattractants, which together draw monocytes, lymphocytes, mast cells, and neutrophils into the arterial wall [14]. The activation of endothelial cells leads to the secretion of monocyte chemoattractant protein-1 (MCP-1), IL-8, intercellular adhesion molecule-1 (ICAM-1), vascular adhesion molecule-1 (VCAM-1), E-selectin, P-selectin, and other inflammatory factors, with lymphocytes and monocytes infiltrating the arterial wall [15]. The effect of two pro-inflammatory cytokines, TNF-alpha and IL-1, which promote the expression of cytokines and adhesion molecules, as well as the migration and mitogenesis of vascular smooth muscle and endothelial cells, is of particular interest [16]. TNF-alpha and IFN-γ have also been associated with the disruption of endothelial cell junctions, leading to leukocyte transmigration, vascular permeability, and matrix degradation, all of which facilitate endothelial damage and atherosclerosis progression [17]. 

### 2.2. Mitochondria 

A number of studies have demonstrated that SARS-CoV-2 infection is associated with impaired mitochondrial function. In preclinical models, SARS-CoV-2 was found to promote the production of mitochondrial reactive oxygen species (mtROS) [12]. The excess of mtROS induces both mitochondrial and cellular damage, resulting in an early senescence of endothelial cells [12]. Furthermore, mtROS activate the transforming growth factor-beta (TGF-β), a known contributor to endothelial-to-mesenchymal transition (EndMT), leading to atherosclerotic plaque rupture and fibrosis [18,19]. In another study, SARS-CoV-2 was proposed to have the ability to directly deregulate mitochondria by localizing its genome very close to the mitochondrial matrix. This allows SARS-CoV-2 to generate double-membrane vesicles (DMVs), a common defensive mechanism of coronaviruses to shield themselves from host cells [20]. DMVs result in impaired viral clearance, leading to the persistence of the virus in pulmonary alveolar cells. Furthermore, mtROS signal pathways for the release of inflammatory cytokines such as interleukins, chemokines, and tissue necrosis factor. SARS-CoV-2, among other viruses, has the potential to induce a massive production of cytokines, known as a cytokine storm [12]. 

### 2.3. Inflammation

Excessive inflammation constitutes a pivotal characteristic in COVID-19 infection, particularly among individuals who progress to severe disease. The onset of a cytokine storm, which refers to a severe immune reaction characterized by the overproduction of cytokines (signaling molecules involved in immune responses) [21], can be triggered not only by viral escape mechanisms but also by genetic defects in the host, such as a deficiency in anti-melanoma differentiation-associated gene 5 (MDA5) leading to reduced interferon levels [22,23]. Cytokines trigger endothelial permeability by harming junctional proteins such as VE-cadherin, or by downregulating their production. CoV-2 has been found to provoke the activation of Toll-like receptor (TLR) 9 and NF-κB, causing an increased expression of inflammatory genes and downregulation of nitric oxide (NO), a major vasodilator and antithrombotic agent [24,25]. SARS-CoV-2 products can also hyperactivate neutrophils via NLR family pyrin domain containing 3 (NLRPR3). Leukocytes produce ROS, extracellular traps (NETs) and proteolytic enzymes, contributing to the perpetuation of inflammation [22,26]. 

The complement system is also a significant part of the immune response. The activation of the complement system can eliminate the pathogen, but it can also damage the ECs which do not express certain protective regulators on their membrane. Additionally, C5a, a protein of the complement cascade, can enhance the chemoattraction of both inflammatory agents and cells. Circulating C3d, C4d, and C5b-9 facilitate thrombus formation. SARS-CoV-2 has been found to upregulate complement markers via the lectin and the alternative pathway [27]. 

### 2.4. Clot Formation

The outbreak of cytokines and systemic vascular injury prompts a procoagulant state [28,29]. Damage to the endothelial glycocalyx, a network of membrane-bound proteoglycans and glycoproteins, results in increased thrombogenic molecules such as von Willebrand Factor (vWF) and P-selectin, leading to intensified platelet aggregation and leukocyte activation [28,30]. Moreover, the disruption of the glycocalyx can reduce the levels of NO and consume anticoagulants such as heparan sulfate proteoglycans, prostacyclin, and tissue factor inhibitor. Cytokine storm also promotes the production of prothrombotic agents, such as thromboxane, plasminogen activator inhibitor-1, and tissue factor. The extended formation of neutrophil extracellular traps (NETs) and antibodies, like antiphospholipid ones, are additional stimuli for coagulation cascade initiation [28]. 

### 2.5. Immune System

A study demonstrated that the number of B cells and T cells, especially natural killer (NK) cells, was lower in patients with severe COVID-19 [22]. Considering the lymphocyte counts, several mechanisms have been proposed, including destruction by the cytokine storm [31], T cell exhaustion [32], a direct infection of T cells [33,34], and virus interference with T cell expansion [35]. Apart from low lymphocytes, the atrophy of lymphoid organs has also been reported. In contrast, monocytes and macrophages are elevated, serving as evidence of heightened inflammatory cytokines such as interleukins (IL), especially IL-1, IL-6, IL-8, and tumor necrosis factor-α [22]. Furthermore, comorbidities related to established vascular injury, such as diabetes or hypertension, as well as predisposing immune system abnormalities, such as advanced age, result in impaired endothelium function and susceptibility in thrombotic events, prolonged hospitalization, and higher mortality [36]. 

## 3. Short-Term Vascular Manifestations and Complications

### 3.1. Thromboembolic Manifestations

**Figure 2:**
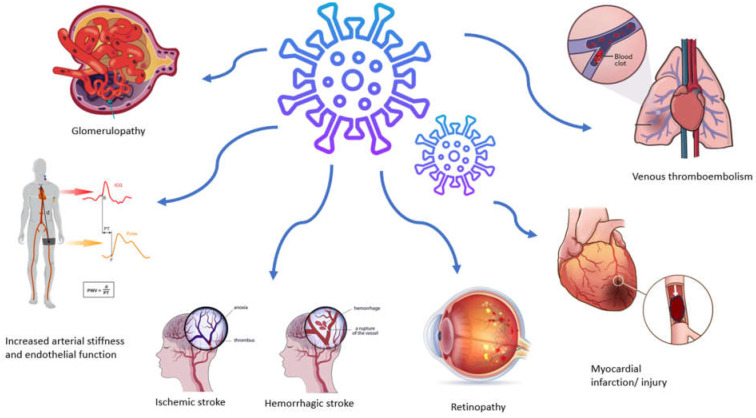
Vascular manifestations and complications associated with SARS-CoV-2 infection.

As mentioned above, SARS-CoV-2 infection induces a prothrombotic and proinflammatory condition that increases the likelihood of a thromboembolic event, with venous thromboembolism being more common than arterial complications (Figure 2) [37]. Large cohort studies have provided evidence showing a heightened occurrence of thrombotic events, reaching rates as high as 47% among patients admitted to intensive care units [38]. Acute myocardial infarction and stroke represent the most common arterial thrombotic (AT) events, while pulmonary embolism, along with lower limb deep vein thrombosis, stands out as the predominant venous thrombotic event [39]. The prevalence and severity of these cases escalate with the increasing severity of the disease, predominantly manifesting in hospitalized and intensive care unit patients or those with comorbidities. Furthermore, the risk of an event persists up to six months after the acute phase [40].

### 3.2. Pulmonary Vessels

Pulmonary involvement is prominent in COVID-19 [41]. ARDS is the result of excessive endothelial dysfunction and continuous cytokine exposure. Proteomic analysis has verified the extended and intense exposure to cytokines in SARS-CoV-2, in contrast to the influenza virus. Pulmonary embolism, with or without concurrent deep vein thrombosis, may manifest in up to 24% of cases [38], alongside the possibility of the occurrence of in situ thrombosis. Vascular enlargement and vascular tree-in-bud sign are common findings in CT studies. Exudative and proliferative diffuse alveolar damage, pneumonia and bronchiolitis, alveolar hemorrhage, and fibrotic areas are present in pathological studies, while microthrombi have been rarely found [9]. Reduced expression levels of ACE2 receptors lead to a decelerated pulmonary dissemination of SARS-CoV-2 [42].

### 3.3. Cardiovascular Alterations

Cardiovascular manifestations were frequent in SARS-CoV-2 patients including acute coronary syndromes, cardiomyopathy, arrhythmias, and cardiogenic shock [43,44]. Elevated troponin among hospitalized individuals was associated with worse outcomes [43]. In a pathological study of 40 hearts after death from COVID-19, myocardial injury was present in 35% of subjects. The main mechanism of injury was proven to be microthrombi, which differ in composition from aspirated thrombi during percutaneous coronary intervention for ST-elevation myocardial infarction in non-COVID-19 patients. These microthrombi had notably elevated fibrin and complement C5b-9 proteins. Coronary artery disease and levels of interleukin-6 were similar among subjects, regardless of the presence of myocardial injury [45]. Elevated non-occlusive fibrin microthrombi and a greater number of involved cardiac arterioles were also the major findings in a study comparing subjects with SARS-CoV-2 and influenza virus [46]. Myocarditis, defined as focal myocyte injury and fibrosis, was also noticed [46]. In another study, viral genome and viral progenies were identified in interstitial cardiac cells or macrophages infiltrating the cardiac tissue, while cardiomyocytes were unaffected. The number of leukocytes (CD3+, CD45+, and CD68) and levels of inflammatory cytokines (IL-6, IL-8, IL-18, TNF-a, interferon-γ, and chemokine ligand 5) were identical in subjects with or without cardiac infection [47]. 

### 3.4. Arterial Stiffness and Endothelial Dysfunction

Progressive stiffening of the aorta enhances the risk of cardiovascular disease and is considered as an independent predictor of mortality [48,49,50]. In a comparative study involving acutely ill patients, higher values of carotid–femoral pulse-wave velocity (cfPWV) and ankle–brachial PWV were observed in patients with COVID-19 compared to controls [51]. Ratchford et al. demonstrated that cfPWV remained elevated 3–4 weeks post-SARS-CoV-2 infection compared to healthy controls [52]. Furthermore, this study highlighted a significantly reduced brachial artery flow-mediated dilation (FMD), indicative of impaired endothelial function [46]. Similarly, a cross-sectional study comparing adults four weeks after positive SARS-CoV-2 testing with healthy controls reported lower brachial artery FMD in the former group, indicating an early-stage vascular dysfunction [52]. Szeghy et al. reported increased carotid stiffness, aortic augmentation index, and Young’s modulus in young adults who had recovered from COVID-19 in comparison to healthy controls [53]. Interestingly, several researchers have reported an increased incidence of arterial hypertension in this patient population; however, larger real-world studies with longer follow-up are required to provide definite answers to this intriguing question [54,55,56,57]. 

### 3.5. Cerebrovasculature

SARS-CoV-2 infection can also affect the brain vasculature causing infarctions of cerebral and cerebellar vessels along with hemorrhagic strokes, but also encephalitis, encephalopathy, demyelinating diseases, and Guillain-Barré syndrome [58,59]. Viral RNA and proteins (nucleocapsid or spike protein) have been detected in cerebrospinal fluid, cerebellum, and cerebrum, indicating a possible transneural proliferation via the ACE2 receptor expressed in terminal nerve cells. Angiotensinogen II enhances macrophages and cytokine storm, leading to the inflammation of cerebral cells. Microgliosis, an infiltration by macrophages, was also recognized in the brainstem. Levels of calcitonin gene-related peptide, a protein with protective abilities against cerebral ischemia, were found to be decreased in COVID-19 patients [60]. Additionally, trans-neuronal transfer is acknowledged as the only way for neurotropic viruses to contaminate the nervous system. Given that olfactory disturbances are very common in COVID-19 and viral RNA and proteins have been isolated from the olfactory nerve, bulb, or cortex, the olfactory nerve has been proposed as an additional viral portal into the central nervous system [61]. In an autopsy study, abundant megakaryocytes were observed in cortical capillaries. These large cells enhance platelet release and adhesion, providing a beneficial substrate for ischemic infarction [62].

### 3.6. Nephropathy

The term COVID-19-Associated Nephropathy (COVAN) has been proposed to describe renal involvement in COVID-19, which according to the American Society of Nephrology (ASN), ranges between 15% to 40% [63]. Acute kidney injury (AKI), proteinuria, and the progression of chronic renal disease have been reported as manifestations of the systemic response to SARS-CoV-2 [64]. Local inflammation, microthrombi, and endothelial injury are common findings even in nephropathy, although studies have been inconsistent about direct viral infection. Histological studies suggest collapsing glomerulopathy and focal segmental glomerulosclerosis in hospitalized patients, while tubular injury is predominately found in critically ill subjects. Collapsing glomerulopathy has been correlated with the presence of high-risk apolipoprotein L1 (APOL1) gene variants [64]. Tubule-interstitial damage is the main mechanism of kidney injury expressed via tubule necrosis, the infiltration of lymphocytes, and the dysfunction of the luminal membrane. Increased CD68+ macrophages, C5b-9 complement particles, and nucleocapsid protein antigen of the virus have been isolated from postmortem autopsies [64]. The dilation of capillary vessels and hyaline thrombi in the microvasculature have also been reported. 

Moreover, the involvement of angiotensin converting enzyme 2 (ACE2) has been thoroughly assessed in studies. ACE2 has been identified as the classic entry receptor of SARS-CoV-2 in the cell. ACE2 metabolizes angiotensin II to angiotensin. The binding of ACE2 and SARS-CoV-2 leads to the downregulation of ACE2 and upregulation of angiotensin II (Ang II). Ang II induces platelet activation, pro-inflammatory cytokine release, and vasoconstriction. This mechanism is common among other morbidities that cause kidney injury, such as diabetes and hypertension. The interruption of renin–angiotensin–aldosterone system (RAAS) inhibitors has been discussed, but it does not seem to alter hospitalization outcome. On the contrary, the extent of kidney injury and prognosis depend on the severity of COVID-19 [65,66]. 

### 3.7. Retinopathy

Retinal vessels express the generic condition of the microvascular system. Retinal microvasculopathy has been observed both in acute and convalescent stages of COVID-19. The main findings in retinographies were hyper-reflective lesions, small hemorrhages, and ischemic type abnormalities like cotton wool spots and retinal sectorial pallor [67]. Optical coherence tomography—angiography (OCTA) has been used to assess the macular capillary vessel density in several studies. Reduced vessel density and inner retinal volume were found both in symptomatic and recovering patients, expressing an impairment of the retinal microcirculation, especially in critically ill individuals. Central and branch retinal vein occlusion has also been described in the literature, regardless of the severity of the disease. On the contrary, central retinal artery occlusion appears rarely [67]. 

### 3.8. Other

Pernio-like lesions (COVID-19 toes) and associated limb ischemia are also diagnosed in COVID-19 [68]. Thrombosis in the mesenteric and portal vasculature have scarcely been reported. In the pediatric population, endothelial dysfunction may lead to a hyperimmune-dysregulatory syndrome known as MIS-C, which shares common features with Kawasaki disease [69].

## 4. Diagnostic Methods and Imaging Techniques

### 4.1. Microvasculature

Nailfold video capillaroscopy (NVC) has been used to visualize microvasculature alterations in COVID-19. NVC is widely used for rheumatic diseases (systemic sclerosis), as well as non-rheumatic diseases (diabetes and hypertension), to analyze changes in microcirculation and endothelial damage. In a study from Italy, multiple lesions were identified by NVC, including dilated and low-density capillaries with asymmetrical orientation and coiled morphology, decelerated blood flow, microthrombosis, and micro-hemorrhages [70]. Of note, the lesions’ extent was correlated with disease severity. Incident darkfield video microscopy and manual video microscopy have also been implemented. OCT and OCTA have been used as non-invasive imaging techniques, to provide a quantitative analysis of retinal and ocular microvascular alterations [70]. 

### 4.2. Macrovasculature

Vessel wall magnetic resonance imaging (vwMRI) has been used in patients with COVID-19 and cryptogenic stroke with unclear etiology. According to Mazzacane et al. [71], vwMRI detected signs of vascular inflammation in the majority of COVID-19 patients with cryptogenic stroke, suggesting the possibility of expanding the diagnostic workup of cryptogenic stroke with vessel wall imaging. Conventional magnetic resonance imaging (MRI), diffusion-weighted image MRI, and MR arteriography/venography have also been used for mapping ischemic infarctions. Computed tomography with or without angiography is widely requested by clinicians for the diagnosis of pulmonary embolism, stroke, or other venous and arterial occlusions. Furthermore, numerous studies have documented an elevated pericoronary fat attenuation index, indicative of increased inflammatory arterial burden, in individuals with a history of COVID-19 infection. This underscores the diagnostic significance of coronary computed tomography angiography and potential future clinical implications within this specific population [72]. The management of acute coronary syndromes in patients with COVID-19 does not seem to differ from the general population, with invasive coronary angiography remaining the gold standard. Positron emission tomography/computed tomography has been utilized to identify the infiltration of inflammatory cells in cardiac and pulmonary tissue, although it is not routinely carried out [73]. Multimodality imaging has been utilized to supply sufficient information for the creation of an algorithm by artificial intelligence, which will enable tissue characterization and an understanding of the vascular implications in COVID-19 [74].

## 5. Potential Therapeutic Interventions 

Patients are categorized as having mild, moderate, or severe illness based on the severity of symptoms, clinical assessments, and imaging findings. Additionally, comorbidities play an important role in the progression of illness, especially those affecting the endothelium, such as diabetes, hypertension, or rheumatic diseases [75,76]. Oxygen therapy, general supportive measures and personalized treatment are mandatory for better outcomes. Several treatment approaches have been tested to mitigate the vascular complications of the disease. 

Anticoagulants, mainly low molecular weight heparins, have been used to prevent thromboembolic events since the early stages of the pandemic, in both hospitalized patients and outpatients [77]. The anticoagulation management of COVID-19 patients has differed across institutions, states, and countries, directly contributing to the diversity in anticoagulation strategies for hospitalized patients [78]. This variability is also evident in clinical trials, where the identification of low, intermediate, and high doses or intensities of anticoagulation differ [79,80,81,82]. Consequently, the lack of well-defined dosing algorithms poses challenges in delivering optimal evidence-based anticoagulation in this clinical context.

Anti-inflammatory drugs, such as steroids, IL-6 inhibitors, interferon α2b, and JAK kinases inhibitors (Baricitinib), have also been widely used in clinical practice for the restriction of the inflammation cascade [83]. Tocilizumab, an IL-6 inhibitor, is indicated for critically ill patients with elevated levels of IL-6 [83]. Furthermore, dexamethasone is recommended for a short period, 3 to 5 days, for patients requiring oxygen therapy [83]. 

Remdesivir, an RNA-dependent RNA polymerase (RdRp) inhibitor, has been shown to effectively inhibit viral replication. In high-risk patients, oral Nirmatrelvir-ritonavir can be administered within 5 days from the disease’s onset [84]. Despite the beneficial effect on mortality in hospitalized patients, evidence suggests a neutral effect on the incidence of AT or VTE events [85]. According to in vitro studies, remdesivir was associated with an increased risk of cardiac arrest (adjusted odds ratio [aOR]: 1.88, 95% confidence interval [CI]: 1.08–3.29) and hypotension (aOR: 1.67, 95% CI: 1.03–2.73) [86]. Monoclonal antibodies, ambavirumab and romisevirumab, can also be given intravenously. Intravenous immunoglobulin (IVIG) and convalescent plasma can be used in high-risk patients and high viral load [84].

Agents targeting the vascular endothelial growth factor (anti-VEGF) and angiopoietin-2 (ANG2), antibodies like Bevacizumab or REGN-COV2, recombinant human ACE2, RNA inhibitors, viral protease inhibitors, viral S-protein inhibitors, small interfering RNAs, quinine derivatives, AT1 and AT2 agonists, and TMPRSS2 serine protease inhibitors are being evaluated in ongoing clinical trials [87]. 

In late 2020 vaccines were added to the prevention quiver. The currently available options include viral vector-based vaccines, inactivated or recombinant protein-based vaccines, nucleic acid-based (DNA or RNA) vaccines, and virus-like particles (VLPs) [88]. A recent meta-analysis of 42 studies assessing the cardiovascular safety of these vaccines reported an increased risk of myocarditis and a neutral effect on the risk of myocardial infarction and arrhythmia events [89]. Nevertheless, the risk of cardiovascular events following SARS-CoV-2 infection far exceeded that observed after vaccination [89]. A comparison of cardiovascular side effects among different types of vaccines is presented in Table 1.

## 6. Long-Term Consequences 

An increasing number of clinical studies suggests that apart from the acute phase of the disease, SARS-CoV-2 may cause prolonged and extended effects on blood vessels. Long-term COVID-19 (LC), characterized by symptoms persisting 12 weeks after the initial infection [90], presents challenges in both diagnosis and treatment due to the limited availability of clinical and laboratory findings, along with the diverse array of symptoms [91,92]. A recent meta-analysis of 41 studies, involving 860,783 patients, identified female sex, older age, higher body mass index, smoking, preexisting comorbidities, and previous hospitalization or ICU admission as risk factors significantly associated with developing long COVID, while also indicating that SARS-CoV-2 vaccination with two doses was associated with a lower risk of long-term implications [93]. Hence, understanding the mechanisms of long-term impacts on the vascular system is essential for implementing the appropriate treatment. 

Several mechanisms previously highlighted as responsible for vascular changes during the acute phase are also implicated in the development of LC. Any exaggerated acute response or the prolonged presence of inflammatory agents may cause cardiovascular complications [94,95]. Acute endothelitis can potentially progress to hypersensitive “leukocytoclastic vasculitis,’’ marked by heightened antibody and auto-antibody production. This exacerbates the existing hyper-coagulant state, resulting in a chronic condition resembling antiphospholipid syndrome, thereby emphasizing the robust connections between hyper-coagulation and inflammation [96,97]. 

SARS-CoV-2 induces chronic inflammation through a combination of mitochondrial dysfunction and the activation of the cyclic GMP-AMP synthase (cGAS)-stimulator of interferon genes (STING) pathway [98,99]. Infected endothelial cells may recognize mitochondrial DNA as a foreign molecule and upregulate the transcription of interferon type I genes. Increased circulating inflammatory agents, endothelial dysfunction, and microthrombosis contribute to an increased risk of developing cardiovascular manifestations. The progression of atherosclerotic plaques, arterial or venous thrombosis, and increased arterial stiffness have been reported [50,51,100,101]. Of note, a study including COVID-19 patients with mild disease reported a widespread and long-lasting pathological process in the vasculature, showing a continual deterioration in arterial stiffness and endothelial function indices over a period of 2–3 months following the infection [96].

A cohort study of fifty individuals with SARS-CoV-2 PCR infection within the last 3 to 6 months reported a reduction in aortic strain and aortic distensibility, coupled with an elevation in pulse pressure (PP) and aortic stiffness index [100]. In a longitudinal investigation conducted by Zanoli et al., forty-one COVID-19 patients demonstrated an increase in aortic PWV at approximately 5 months post-infection [101]. Although this measure of arterial stiffness notably decreased, it remained higher compared to controls even 1 year after COVID-19 infection [101]. The persistence of impairment in arterial stiffness indices was further affirmed by Lambadiari et al. [102], who showed higher cfPWV, central pulse pressure (PP), and systolic blood pressure (BP) among seventy COVID-19 patients 4 months post-diagnosis.

Aortic aneurysms may experience an accelerated rate of progression, and hence an increased risk of rupture in the context of LC-associated hyper-coagulation [96]. A proposed mechanism implicates vasa vasorum thrombosis, leading to an accelerated growth of the intraluminal thrombus [96]. This thrombus tightly adheres to the aneurysm walls, exacerbating parietal hypoxia [96]. Consequently, a substantial release of oxygen reactive species and elevated oxidative stress occurs, further harming the aneurysm walls already undergoing an inflammatory process associated with atherosclerosis [103,104]. In the context of COVID-19, the circulating inflammatory cells specific to the virus infiltrate the aneurysm thrombus, making contact with foreign molecules within the aortic atherosclerotic plaques, such as cholesterol crystals and calcium compounds [105]. This interaction amplifies the local inflammatory response, leading to the release of elastases and metalloproteinases [105]. These proteases, in turn, facilitate the breakdown of collagen and elastin components in the aortic medial tunica, promoting the proliferation of smooth muscle cells and accelerating their apoptosis [105]. 

Coronary arteries are also anticipated to exhibit a comparable LC pathology, indicating an increased risk of obstruction with significant prognostic implications [106]. Of note, a recent study employing computed tomography revealed that COVID-19 patients with elevated coronary artery calcification (CAC) scores experienced more adverse prognosis [107]. This suggests that calcific atheromasia of the coronary arteries in individuals with COVID-19 could serve as an important prognostic indicator for unfavorable clinical outcomes [107]. Moreover, a recent meta-analysis demonstrated an increased risk for sleep disturbances during the COVID-19 pandemic [108], which inevitably leads to a disruption in circadian rhythm. This disruption has been suggested as an additional potential factor contributing to the induction of vascular calcification [109].

Although the effects of COVID-19 on the vasculature have been thoroughly examined during the acute phase, there is currently a lack of data regarding these effects in LC. Understanding the long-term impact of COVID-19 is vital for identifying individuals experiencing LC, guiding clinical management, and averting potential severe vascular complications in future patients. To this end, further research is necessary [110]. 

## 7. Prevention and Future Directions

Fundamental strategies for mitigating severe vascular complications encompass the prompt detection and management of high-risk individuals, along with a focus on anticoagulation therapy as warranted. Initiating antiviral medications early in the course of the disease, combined with vigilant monitoring of hospitalized patients, holds promise in potentially ameliorating adverse outcomes and enhancing morbidity and mortality. Nevertheless, the precise patient subgroups that would derive greater benefit from the timely initiation of these antiviral therapies remain incompletely understood and necessitate further investigation. Widespread immunization against SARS-CoV-2 through vaccinations can also contribute to limiting the extent of vascular alterations induced by the virus. Future research endeavors aim for innovative treatments targeting inflammatory factors and safeguarding endothelial function. The early identification and comprehensive assessment of long COVID patients offer valuable insights for a deeper understanding of the enduring consequences of the virus. Numerous ongoing trials assess the effectiveness of treatments originally designed for other endothelial-affecting conditions in the context of COVID-19, with some already transitioning into clinical practice. It is imperative to highlight that individuals with pre-existing conditions should receive timely medication for the management of underlying hematological disorders, hypertension, and diabetes. The progressive increase in patients’ age is associated with the accumulation of comorbidities and pre-existing endothelial damage, posing challenges in their management and therapeutic interventions. Despite advances in medicine, health systems worldwide appeared unprepared to deal with SARS-CoV-2, a virus that mostly affects the vascular system. Therefore, further research on the area of vascular alterations is essential, not only for designing new treatments for COVID-19 but also to expand our knowledge of the underlying pathophysiological mechanisms triggering systemic responses in the body, paving the way for novel approaches and improved management strategies.

## 8. Conclusions

In summary, COVID-19 has a significant impact on multiple organs, primarily through its effects on the vascular system, leading to diverse manifestations and complications. COVID-19 induces intracellular damage, including mitochondrial dysfunction, massive cytokine production, microthrombosis, and immune system abnormalities, culminating in a prothrombotic state and systemic vascular injury. Patients present with various clinical complications, such as thromboembolic events, cardiovascular and cerebrovascular manifestations, nephropathy, and retinopathy, often associated with severe illness or comorbidities. Several imaging techniques, histopathological studies, and diagnostic tools like video capillaroscopy and magnetic resonance imaging have been employed to visualize and study vascular alterations in these patients. Beyond the acute phase, SARS-CoV-2 infection can lead to persistent effects on blood vessels, known as long-term COVID-19. However, considering that COVID-19 infection entails a continuum of pathophysiological changes, a sterilized classification into short and long-term changes may appear simplistic. Early detection, appropriate medication and management, anticoagulation therapy, and vaccinations can substantially limit adverse events and improve clinical outcomes. Ongoing research aiming at innovative treatments targeting inflammation and endothelial agents is crucial in preventing severe vascular complications. 

## Figures and Tables

**Table 1:** Comparison of cardiovascular adverse effects across different vaccine platforms.

Variables	mRNA	Adenoviral Vector	Inactivated Whole Virus	Protein Subunit
Part A				
Brands	Moderna’s Spikevax, Pfizer/BioNTech’s Comirnaty	Oxford/AstraZeneca’s Vaxzevria, India Serum Institute’s Covishield, Johnson & Johnson’s Janssen, and CanSino’s Convidecia	Bharat Biotech’s Covaxin, Sinopharm’s Covilo, and Sinovac’s CoronaVac	Novavax’s Nuvaxovid and India Serum Institute’s COVOVAX
Mechanisms	Encodes the spike protein of SARS-CoV-2	Packages the SARS-CoV-2 coding sequence in a recombinant adenovirus	Inactivated SARS-CoV-2 with adjuvant	Contains isolated and purified SARS-CoV-2 proteins
Part B. Pooled vaccine effectiveness [89]				
Infection	3 doses: 96%	2 doses + 1 dose mRNA: 88%	2 doses: 57%	-
	2 doses: 77%	2 doses: 74%		
	1 dose: 59%	1 dose: 61%		
Symptomatic infection	3 doses: 98%	1 dose: 43%	2 doses: 72%	-
	2 doses: 91%		1 dose: 48%	
	1 dose: 55%			
Severe infection	2 doses: 99%	2 doses: 96%	2 doses: 88%	-
	1 dose: 96%		1 dose: 66%	
Hospital admission	3 doses: 95%	2 doses: 81%	-	-
	doses: 81%	dose: 80%		
Cardiovascular side effects	Myocarditis, pericarditis, acute myocardial infarction, arrhythmia, stress cardiomyopathy, thrombosis, thrombocytopenia	Myocarditis, pericarditis, acute myocardial infarction, thrombosis, thrombocytopenia	Type 1 Kounis syndrome	-

Part A: Describes the different available vaccine platforms (mRNA, adenoviral vector, inactivated whole virus, and protein subunit) and the mechanisms of immunization. Part B: Summarizes the effectiveness of each vaccine platform in decreasing the likelihood of (i) any infection, (ii) symptomatic infection, (iii) severe infection, and (iv) hospital admission after receiving 1, 2, or 3 doses of vaccination. The final row outlines the most commonly reported cardiovascular side effects associated with each vaccine platform. Kounis syndrome manifests as acute coronary syndrome, triggered by an allergic or immune response to a drug or other substance. Abbreviations: SARS-CoV-2, severe acute respiratory syndrome coronavirus 2.

## Data Availability

This is a review article analyzing secondary data, so data sharing is not applicable.
